# Atopic dermatitis and chronic sinusitis: a two-sample Mendelian randomized study

**DOI:** 10.1016/j.bjorl.2026.101789

**Published:** 2026-03-09

**Authors:** Jian Luo, Yuanzhi Zhu, Yuting Zhang, Kai Zhang

**Affiliations:** The First People’s Hospital of Yibin, Department of Otorhinolaryngology, Yibin, Sichuan, China

**Keywords:** Atopic dermatitis, Chronic sinusitis, Causal relationship, Mendelian randomization study

## Abstract

**Objectives:**

Despite observational studies have indicated a potential association between Atopic Dermatitis (AD) and Chronic Sinusitis (CRS), it is uncertain whether there is a causal relationship between AD and CRS. To address this ambiguity, a two-sample bidirectional Mendelian randomization methodology was utilized to examine the potential causal association between AD with CRS.

**Methods:**

The summary data for the Genome-Wide Association Study (GWAS) on AD and CRS were sourced from the FinnGen consortium. To investigate the causal link between AD and CRS, we applied several approaches, including Inverse-Variance Weighting (IVW), MR-Egger regression, weighted median, simple mode, and weighted mode methods. Furthermore, the heterogeneity, horizontal pleiotropy, and sensitivity were investigated using several methods.

**Results:**

Based on the random-effect IVW approach, results showed a 15.8% increased risk of CRS in those with AD compared with non-AD populations (p = 7.771e-07, OR = 1.158, 95% CI 1.092–1.227). Inversely, there was no direct evidence that CR increased the risk of AD (p = 0.979, OR = 1.001, 95% CI 0.908–1.104). The MR-Egger intercept test results indicated an absence of horizontal pleiotropy in this study (AD: p = 0.381; CRS: p = 0.144). Additionally, the leave-one-out test demonstrated the results of both forward and backward analyses were reliable and robust.

**Conclusion:**

Our study confirmed the causal effect of AD on CRS, with AD increasing the risk of developing CRS. Conversely, CRS did not affect the risk of developing AD. Our study suggested that in the prevention and treatment of CRS, physicians cannot ignore the impact of AD on CRS.

**Level of evidence:**

Level 2.

## Highlights

• Mendelian analysis reveals genetic association between AD and CRS.
• No reverse genetic association detected from CRS toward AD.
• Results support shared inflammatory pathways in AD and CRS.

## Introduction

Chronic Sinusitis (CRS) represents a persistent inflammatory disorder affecting the mucosa of the paranasal sinuses; The clinical manifestations of CRS are characterized by two or more symptoms, the main symptom is nasal obstruction or nasal discharge, and the secondary symptoms are facial pain or pressure, decreased or loss of smell, and at least one of the main symptoms is included; The symptoms of CRS lasting ≥12-weeks with evidence of sinus inflammation confirmed by nasal endoscopy or sinus CT.^1^ CRS is categorized into two subtypes: CRS with Nasal Polyps (CRSwNP) and CRS without Nasal Polyps (CRS sNP), depending on whether nasal polyps are present. CRS is a common disease in most parts of the world, affecting 5%–12% of the total population and placing a heavy burden on society in terms of healthcare consumption and lost productivity.^1^, ^2^, ^3^ To date, multiple epidemiological studies have consistently shown that only asthma and increasing age are risk factors for CRS.^1^^,^^4^^,^^5^ To further reduce the burden of preventing and treating CRS, more attention should be paid to other potentially modifiable risk factors, such as Atopic Dermatitis (AD).

Atopic dermatitis represents a persistent, recurrent inflammatory disorder affecting the skin. The clinical manifestations of AD are varied, with the most basic features being dry skin, chronic eczematous lesions, and obvious eczema.^6^ Over the past twenty years, the incidence of AD has escalated dramatically, positioning it as the leading contributor to the burden of non-fatal diseases. A study from Africa indicates a gradual rise in AD prevalence across sub-Saharan Africa, where rates now exceed 15% among children.^7^ The Global Burden of Disease Study estimates AD prevalence to be between 15% and 20% in children and up to 10% in adults.^8^ Recent research indicates that AD increases the risk of several diseases.^9^, ^10^, ^11^ However, there is no literature report on whether AD can cause CRS. Chandra et al.^12^ documented a 7% prevalence of AR in individuals diagnosed with AD. CRS prevalence in the general population ranged from 3% to 10.9%.^13^^,^^14^ Atopic dermatitis does not seem to affect the prevalence of CRS. An observational study found that young and middle-aged CRS patients has a higher risk of developing AD, but this observational study was insufficient to prove that CRS is a risk factor for AD.^15^ Consequently, as of now, the causal relationship between AD and CRS remains unclear.

The most robust approach to determine whether a causal relationship exists between two variables is through the implementation of a Randomized Controlled Trial (RCT). Nonetheless, the execution of a clinical RCT requires substantial resources, including considerable manpower, time, and financial investment, and is difficult to carry out. Mendelian Randomization (MR) is an emerging epidemiological approach that constructs models and infers and evaluates causal effects by retrieving Single Nucleotide Polymorphisms (SNPs) that are strongly associated with exposure as instrumental variables.^16^ Since alleles are randomly distributed during human gamete formation, the MR method can overcome biases such as reverse causality and confounding factors. Recent advancements in GWAS have led to the accumulation of tens of thousands, or even millions, of associations between genetic variants and phenotypic traits. Using published GWAS data for MR research can not only make causal inferences but also save a lot of research costs, so MR is widely used by researchers.

In this research, we conducted a bidirectional two-sample MR analysis using GWAS statistical summary data for AD and CRS to examine the potential causal link between them. As far as we know, this study represents the first MR analysis aimed at exploring this potential causal relationship.

## Methods

### Study design and data sources

**Fig. 1:**
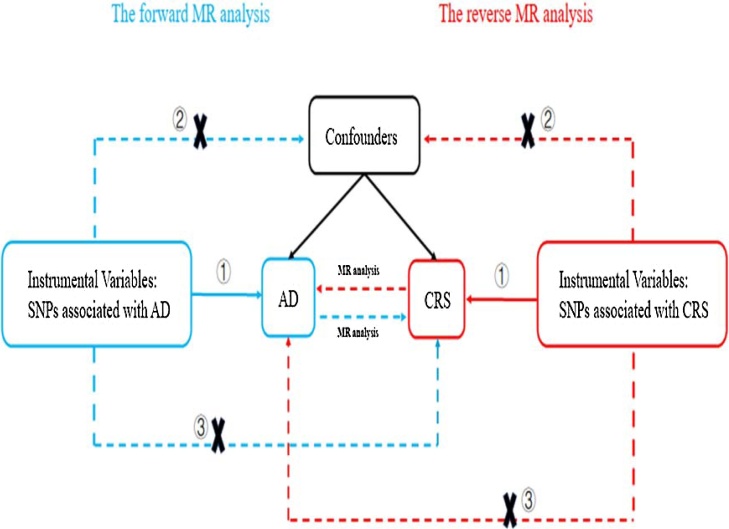
Description of this bidirectional MR study design: (1) Genetic variants are associated with exposure; (2) Genetic variants are independent of confounders; (3) Genetic variants affect outcome only through risk factor.

Fig. 1 briefly described the two-sample MR study process between AD and CRS. To assess causal links between AD and CRS, a bidirectional two-sample MR analysis was conducted. SNPs were identified as Instrumental Variables (IVs) for subsequent analysis. The MR design needs to fulfill three essential assumptions: (1) The selected SNPs exhibit a strong association with exposure (p < 5*10^−8^, LD < 0.001, *F* > 10); (2) The selected SNPs should be independent of any confounding factors that might influence the relationship between exposure and outcome; (3) The selected SNPs affect the outcome only by affecting the exposure.^17^

The summary data for AD from GWAS was sourced from the FinnGen Consortium (R11 version). The AD data set included 421,381 individuals, including 26,905 cases and 394,476 controls.^18^ The GWAS summary data of CRS also came from the FinnGen Consortium (R11 version). The CRS data set included 357,737 individuals, including 19,856 cases and 337,881 controls.^18^ Each participant was from Europe and has the same genetic background, so the inference of causality will not be affected by the ancestry factor. All of these original studies have obtained ethical approval. All participants provided informed consent, with the original consent forms collected and securely retained by the primary researchers.

### Instrumental variable selection

Conducting an MR study requires the following three assumptions: association assumption, independence assumption, and exclusion assumption.^17^ Therefore, the selected instrumental variables must simultaneously meet the following criteria: (1) p-value associated with exposure < 5*10^−8^, and *F* > 10; (2) To ensure the independence of IVs, we implemented the clumping process (*r*^2^ < 0.001, physical window = 10,000 kb) to exclude linkage disequilibrium; (3) Exclude palindromic SNPs with intermediate allele frequencies; (4) Exclude SNPs associated with confounders and outcomes.

### Statistical methods

Five different methods were employed to explore the causal relationship between AD and CRS: Inverse Variance Weighting (IVW), MR-Egger regression, weighted median, simple model, and weighted model methods. In our MR analysis, IVW was utilized as the primary method to evaluate the causal association between AD and CRS because IVW assumes that all SNPs used in this MR study are true genetic instruments and therefore can produce the most accurate estimates.^19^ Cochrane's *Q* test was employed to evaluate the heterogeneity of individual SNPs. When the p-value in this MR result was below 0.05, we applied the IVW method with random-effects model to evaluate the causal relationship between AD and CRS.^20^ The MR-Egger intercept test was performed to assess whether horizontal pleiotropy was present, with a p-value below 0.05 indicating its existence.^21^ To test the stability of the results, we employed the leave-one-out method. This method was used to assess whether the overall effect was affected by a single SNP. The causal relationship of the exposure to the outcome was characterized using the Odds Ratio (OR) and the 95% Confidence Interval (95% CI). R software version 4.4.1, *R* Studio, and TwoSampleMR package (version 0.5.10) were used to select instrumental variables, perform statistical analysis, and visualize the results. For all statistical evaluations, two-tailed testing procedures were implemented, with a p-value of less than 0.05 deemed statistically significant.

## Results

### Atopic dermatitis to chronic sinusitis

According to the instrumental variable selection criteria, 62 SNPs were finally identified as instrumental variables for testing the causal effect of AD on CRS. Detailed information about the SNPs was shown in Supplementary Table S1.

The heterogeneity of individual SNPs was evaluated by the Cochrane's *Q* test. Given that the p-value was 4.150e-08, this research utilized the random-effects IVW approach to assess the causal influence of AD on CRS. The results suggested that AD serves as an independent risk factor for CRS development (p = 7.771e-07, OR = 1.158, 95% CI 1.092–1.227). While heterogeneity is present, it does not impact the results of the IVW analysis. At the same time, the results obtained by weighted median, simple mode, and weighted mode methods aligned with those derived from the IVW method. The results from the five methods employed in this MR analysis are presented in Table 1. The MR-Egger intercept test revealed no evidence of horizontal pleiotropy in the association (p = 0.381). Furthermore, the leave-one-out test showed that no single SNP affected the results of our MR analysis (Supplementary Fig. S1). In summary, this result was reliable and robust.

**Table 1:** The MR results by five methods.

Exposure	Outcome	SNP(n)	Method	β	se	OR	OR95%CI		P-value
Atopic dermatitis	Chronic sinusitis	62	MR Egger	0.074	0.088	1.077	0.907	1.278	4.031e-01
			Weighted median	0.161	0.033	1.174	1.100	1.254	1.500e-06
			Inverse variance weighted	0.147	0.030	1.158	1.092	1.227	7.771e-07
			Simple mode	0.229	0.094	1.257	1.046	1.511	1.770e-02
			Weighted mode	0.232	0.083	1.261	1.072	1.483	6.802e-03

### Chronic sinusitis to atopic dermatitis

According to the instrumental variable selection criteria, 18 SNPs were finally determined as instrumental variables for testing the causal effect of CRS on AD. Detailed information about SNPs was shown in Supplementary Table S2.

The heterogeneity of individual SNPs was evaluated by the Cochrane's *Q* test. Given that the p-value was 3.629e-06, this research utilized the random-effects IVW approach to assess the causal influence of CRS on AD. The results showed that CRS does not increase the likelihood of developing AD (p = 0.979, OR = 1.001, 95% CI 0.908–1.104). While heterogeneity is present, it does not impact the results of the IVW analysis. At the same time, the results obtained by MR-Egger, simple model aligned with those derived from the IVW method. The results from the five methods employed in this MR analysis are presented in Table 2. The MR-Egger intercept test revealed no evidence of horizontal pleiotropy in the association (p = 0.144). Furthermore, the leave-one-out test showed that no single SNP affected the results of our MR analysis (Supplementary Fig. S2). In summary, this result is reliable and robust.

**Table 2:** The MR results by five methods.

Exposure	Outcome	SNP(n)	Method	β	se	OR	OR95%CI		P-value
Chronic sinusitis	Atopic dermatitis	18	MR Egger	−0.251	0.171	0.778	0.556	1.088	0.162
			Weighted median	−0.089	0.042	0.915	0.843	0.993	0.034
			Inverse variance weighted	0.001	0.050	1.001	0.908	1.104	0.979
			Simple mode	−0.1001	0.051	0.904	0.818	1.000	0.066
			Weighted mode	−0.101	0.044	0.904	0.830	0.985	0.034

## Discussion

A cohort study including 161 patients showed that 15% of CRSsNP patients and 11% of CRSwNP patients were confirmed to have AD; in contrast, only 4% of patients in the control group had AD.^22^ An observational study showed that a 7% prevalence of CRS among patients diagnosed with AD.^12^ In the general population, the prevalence of CRS ranged from 3% to 10.9%.^13^^,^^14^ AD may not affect the prevalence of CRS in patients. Observational studies cannot make causal inferences between two variables. This study utilized a bidirectional two-sample MR analysis to examine the causal link between AD and CRS. The results showed that AD can cause the development of CRS and AD serves as a risk factor for CRS. In addition, through comprehensive reverse MR analysis, we found no evidence of a reverse causal link between AD and CRS, which effectively eliminated any effect of CRS on AD.

Some potential reasons may explain why AD act as a risk factor for CRS. On the one hand, the activation of the Th2 axis immune response in AD leads to the secretion of diverse cytokines, including IL-4, IL-5, IL-13, and IL-31.^23^ Previous research has demonstrated both IL-4 and IL-13 are capable of inducing the expression of periostin, which can bind to proteins involved in fibrotic reactions and promote the occurrence of sinusitis.^24^ Studies have found that eotaxin^25^ and eosinophil cationic protein^26^ are highly expressed in patients with AD. In CRS with nasal polyps, elevated levels of IL-5, eosinophil cationic protein, and eotaxin work in concert to regulate the chemotaxis, activation, and survival of eosinophils.^27^, ^28^, ^29^, ^30^ Activated eosinophils release the contents of their granules, causing tissue damage and contributing to the progression of sinusitis.^31^ On the other hand, studies have found that the Th1 immune response is implicated in the chronic stage of AD.^23^ The activation of the Th1 axis immune response in chronic AD results in the secretion of various cytokines, including Interferon-γ (IFN-γ).^32^^,^^33^ Studies have found that IFN-γ reduces the epithelial barrier function of sinus epithelial cells, thereby causing sinusitis.^34^

Our study has several important advantages. First, this study represents the first bidirectional two-sample MR study of the causal relationship between AD and CRS, and Mendelian randomization is closest to RCT studies, saving research costs. Mendelian randomization studies offer an advantage over traditional observational studies by addressing key limitations, such as reverse causality, confounding factors, and various biases. Second, instrumental variables that were strongly associated with AD were screened in the forward MR analysis, and instrumental variables that were strongly associated with CRS were screened in the reverse MR analysis (*F* statistic ≥10) to ensure the validity of the research results. Finally, to assess horizontal pleiotropy, the MR-Egger intercept test was conducted, while a leave-one-out analysis was performed to evaluate the sensitivity of the results. The findings suggested that the causal association between AD and CRS remained both consistent and reliable across various analytic approaches.

Nevertheless, our study has several limitations. First, given that all participants were of European descent, our findings may not be applicable to individuals of other ancestries. Second, due to the limitations of GWAS data, we only evaluated the causal relationship between AD and CRS but did not distinguish between CRSwNP and CRSsNP. Finally, although our study indicated a potential causal relationship between AD and CRS, due to the limitations of the existing data, this analysis cannot provide evidence for the specific mechanism of CRS occurrence.

## Conclusion

We confirmed a causal effect of AD on CRS, with AD increasing the risk of CRS. Conversely, there is no evidence that CRS influence the risk of AD. Therefore, the impact of AD should be considered in the prevention and treatment of CRS. Nonetheless, further investigation of genetic variation in CRS is needed to confirm the genetic causal effects found in our study.

## ORCID ID

Yuanzhi Zhu: 0000-0002-7928-9892

Yuting Zhang: 0009-0008-1573-1769

Kai Zhang: 0000-0003-4860-368X

## Funding

No.

## Data availability statement

The authors declare that all data are available in repository.

## Declaration of competing interest

The authors declare no conflicts of interest.
