# Supplementary material for: Atopic dermatitis and chronic sinusitis: a two-sample Mendelian randomized study
Source: Braz J Otorhinolaryngol. 2026 Mar 9;92(3):101789. doi: 10.1016/j.bjorl.2026.101789 (PMC12994028; doi:10.1016/j.bjorl.2026.101789)
Supplement: Supplementary file 1 [file mmc1.docx]

**BJORL-D-25-00087_Supplementary Material**

**Supplementary Table 1** Detailed information of selected SNPs for MR analysis of the causal effect of AD on CRS.

| SNP | Effect allele | Other  allele | AD | | | | CRS | | | |
| --- | --- | --- | --- | --- | --- | --- | --- | --- | --- | --- |
|  |  |  | eaf | β | se | P-value | eaf | β | se | *P*-value |
| rs10195800 | T | C | 0.241012 | 0.0607393 | 0.010571 | 9.16E-09 | 0.240727 | 0.003617 | 0.012184 | 0.76657 |
| rs1026788 | C | T | 0.55907 | 0.0617531 | 0.0092 | 1.91E-11 | 0.559871 | 0.00943 | 0.010489 | 0.368661 |
| rs10791824 | G | A | 0.635642 | 0.102996 | 0.009538 | 3.49E-27 | 0.63571 | 0.026944 | 0.010816 | 0.012731 |
| rs1107943 | C | T | 0.074708 | 0.0940082 | 0.017018 | 3.31E-08 | 0.075099 | 0.023018 | 0.019752 | 0.243881 |
| rs11156875 | G | A | 0.181361 | 0.0761642 | 0.011646 | 6.16E-11 | 0.181198 | 0.019791 | 0.013506 | 0.142805 |
| rs11236813 | C | G | 0.116803 | -0.110218 | 0.014437 | 2.27E-14 | 0.117105 | -0.00598 | 0.016138 | 0.710911 |
| rs112502960 | A | G | 0.425534 | 0.0891834 | 0.009146 | 1.82E-22 | 0.424598 | 0.04843 | 0.010467 | 3.72E-06 |
| rs116674320 | A | G | 0.126334 | -0.0768547 | 0.013857 | 2.92E-08 | 0.126289 | -0.03314 | 0.01563 | 0.033999 |
| rs117137535 | A | G | 0.03965 | 0.157326 | 0.022554 | 3.05E-12 | 0.039768 | 0.061318 | 0.026696 | 0.021626 |
| rs117710327 | A | C | 0.100247 | -0.111426 | 0.015625 | 9.93E-13 | 0.100093 | -0.07633 | 0.017683 | 1.58E-05 |
| rs11949727 | A | G | 0.160749 | -0.0745463 | 0.012579 | 3.10E-09 | 0.160659 | -0.02766 | 0.014158 | 0.050722 |
| rs12123821 | T | C | 0.03912 | 0.292812 | 0.021753 | 2.66E-41 | 0.038974 | -0.01948 | 0.02686 | 0.468275 |
| rs12644693 | G | C | 0.284531 | 0.0580169 | 0.010023 | 7.10E-09 | 0.284303 | 0.01958 | 0.011526 | 0.089353 |
| rs13275219 | C | T | 0.680359 | -0.0658936 | 0.009689 | 1.04E-11 | 0.680909 | -0.02164 | 0.01114 | 0.052007 |
| rs141945528 | C | A | 0.007156 | 0.335627 | 0.049351 | 1.04E-11 | 0.006922 | -0.04383 | 0.063031 | 0.486817 |
| rs142185235 | A | C | 0.057441 | 0.188418 | 0.018539 | 2.89E-24 | 0.057639 | -0.00359 | 0.022225 | 0.871738 |
| rs142841116 | T | C | 0.074716 | 0.102165 | 0.016957 | 1.69E-09 | 0.07458 | 0.020893 | 0.019954 | 0.295084 |
| rs145035369 | G | A | 0.031203 | -0.148614 | 0.026974 | 3.60E-08 | 0.031009 | 0.036091 | 0.029899 | 0.227401 |
| rs1504215 | A | G | 0.238405 | -0.103476 | 0.010835 | 1.30E-21 | 0.238712 | -0.05101 | 0.012271 | 3.22E-05 |
| rs1727326 | G | C | 0.855103 | 0.0733076 | 0.013146 | 2.46E-08 | 0.854395 | 0.002085 | 0.014773 | 0.887749 |
| rs17371133 | C | A | 0.427855 | 0.0959478 | 0.009215 | 2.19E-25 | 0.427808 | 0.010926 | 0.010573 | 0.301444 |
| rs17881320 | T | G | 0.079814 | 0.101947 | 0.016386 | 4.92E-10 | 0.079529 | 0.056513 | 0.018836 | 0.002697 |
| rs182568416 | T | G | 0.0287 | 0.160521 | 0.026171 | 8.60E-10 | 0.028685 | 0.035976 | 0.031314 | 0.2506 |
| rs2041733 | C | T | 0.468233 | -0.0823953 | 0.009162 | 2.41E-19 | 0.469287 | -0.01828 | 0.010435 | 0.079875 |
| rs2391683 | G | T | 0.717762 | -0.060683 | 0.010058 | 1.60E-09 | 0.717447 | 0.006334 | 0.011563 | 0.583852 |
| rs245479 | G | A | 0.751125 | 0.0607148 | 0.010617 | 1.07E-08 | 0.751788 | -0.01431 | 0.012046 | 0.234969 |
| rs2893907 | C | A | 0.699388 | 0.0742469 | 0.010046 | 1.46E-13 | 0.699571 | 0.025659 | 0.011369 | 0.024009 |
| rs2967676 | C | A | 0.221423 | 0.0729911 | 0.010826 | 1.56E-11 | 0.221363 | -0.00996 | 0.012522 | 0.426195 |
| rs34290285 | A | G | 0.216587 | -0.0691468 | 0.011188 | 6.40E-10 | 0.216847 | -0.05029 | 0.012745 | 7.95E-05 |
| rs350143 | C | T | 0.723395 | 0.0683677 | 0.010297 | 3.15E-11 | 0.723482 | -0.00795 | 0.011667 | 0.495712 |
| rs35570272 | T | G | 0.355224 | 0.0694384 | 0.009473 | 2.30E-13 | 0.355548 | -0.00179 | 0.010879 | 0.868969 |
| rs3861950 | C | T | 0.318837 | 0.0627485 | 0.009685 | 9.23E-11 | 0.318358 | 0.015816 | 0.011139 | 0.155651 |
| rs4821563 | C | A | 0.736251 | 0.0642517 | 0.010427 | 7.17E-10 | 0.736499 | -0.01737 | 0.011796 | 0.140751 |
| rs4851008 | C | G | 0.80956 | -0.130965 | 0.011356 | 8.99E-31 | 0.809217 | 0.002553 | 0.013249 | 0.847204 |
| rs501764 | T | G | 0.829355 | -0.0757116 | 0.011955 | 2.40E-10 | 0.828862 | -0.03063 | 0.013793 | 0.026349 |
| rs56094005 | G | A | 0.046418 | -0.170139 | 0.022758 | 7.66E-14 | 0.046381 | 0.008468 | 0.025019 | 0.735001 |
| rs56121811 | T | C | 0.391324 | 0.0581945 | 0.009333 | 4.51E-10 | 0.390941 | 0.000906 | 0.010694 | 0.93248 |
| rs58568327 | A | G | 0.176289 | -0.0897167 | 0.012186 | 1.81E-13 | 0.176385 | 0.002427 | 0.013725 | 0.859665 |
| rs6062490 | C | G | 0.809852 | 0.11692 | 0.011839 | 5.28E-23 | 0.809423 | 0.026582 | 0.013223 | 0.044411 |
| rs61816766 | C | T | 0.007766 | 0.349666 | 0.047796 | 2.56E-13 | 0.00768 | 0.000121 | 0.060356 | 0.998403 |
| rs61839660 | T | C | 0.040584 | 0.144308 | 0.022394 | 1.16E-10 | 0.04087 | -0.05286 | 0.026361 | 0.044919 |
| rs62089050 | T | C | 0.100668 | 0.0832234 | 0.014786 | 1.82E-08 | 0.100474 | 0.008307 | 0.017289 | 0.630869 |
| rs62160676 | C | T | 0.372228 | 0.0550283 | 0.009791 | 1.91E-08 | 0.370267 | -0.01175 | 0.011244 | 0.296079 |
| rs6796 | C | T | 0.32162 | -0.0571138 | 0.009838 | 6.42E-09 | 0.322192 | -2.31E-05 | 0.011173 | 0.998352 |
| rs6852559 | A | G | 0.138985 | 0.0710302 | 0.013029 | 4.99E-08 | 0.139207 | 0.012191 | 0.015052 | 0.417969 |
| rs6996614 | A | C | 0.567801 | 0.0760152 | 0.009228 | 1.76E-16 | 0.568523 | 0.003348 | 0.010518 | 0.750234 |
| rs73018933 | A | G | 0.247323 | -0.076101 | 0.010678 | 1.02E-12 | 0.24745 | -0.02774 | 0.012054 | 0.021359 |
| rs73068668 | A | G | 0.08225 | -0.0924758 | 0.016913 | 4.56E-08 | 0.082765 | -0.02837 | 0.018893 | 0.133233 |
| rs73222673 | A | G | 0.126468 | 0.0777905 | 0.01357 | 9.88E-09 | 0.126616 | 0.004393 | 0.015748 | 0.78027 |
| rs7688384 | T | A | 0.035906 | -0.148156 | 0.025282 | 4.62E-09 | 0.035778 | -0.00855 | 0.027901 | 0.759186 |
| rs7705653 | T | C | 0.311792 | -0.0715805 | 0.009908 | 5.03E-13 | 0.312148 | -0.00279 | 0.011225 | 0.803504 |
| rs7931483 | G | A | 0.192169 | 0.0698101 | 0.011433 | 1.02E-09 | 0.191506 | -0.02692 | 0.01325 | 0.042194 |
| rs847 | A | C | 0.417189 | 0.119955 | 0.009177 | 4.85E-39 | 0.416382 | 0.030893 | 0.010537 | 0.003369 |
| rs911263 | C | T | 0.635415 | -0.115437 | 0.009408 | 1.31E-34 | 0.635396 | -0.02848 | 0.010806 | 0.008401 |
| rs9265981 | T | C | 0.690541 | -0.0601827 | 0.009763 | 7.06E-10 | 0.690286 | -0.0099 | 0.011222 | 0.377497 |
| rs9277376 | C | A | 0.258776 | 0.0678805 | 0.010333 | 5.04E-11 | 0.258886 | 0.014192 | 0.011887 | 0.232501 |
| rs9310555 | C | G | 0.250805 | 0.0581733 | 0.010375 | 2.06E-08 | 0.250144 | 0.011771 | 0.011969 | 0.325381 |
| rs943451 | A | G | 0.544291 | -0.0558629 | 0.009114 | 8.82E-10 | 0.544546 | -0.00346 | 0.010426 | 0.73991 |
| rs964293 | C | T | 0.698876 | -0.0709756 | 0.009899 | 7.51E-13 | 0.699255 | -0.02595 | 0.011399 | 0.02282 |
| rs9859579 | A | C | 0.324264 | -0.0596372 | 0.009814 | 1.23E-09 | 0.324235 | -0.01747 | 0.011165 | 0.117574 |
| rs987106 | A | G | 0.112824 | -0.113411 | 0.014709 | 1.26E-14 | 0.113307 | 0.038635 | 0.016437 | 0.01875 |

**Supplementary Table 2** Detailed information of selected SNPs for MR analysis of the causal effect of CRS on AD.

| SNP | Effect allele | Other  allele | CRS | | | | AD | | | |
| --- | --- | --- | --- | --- | --- | --- | --- | --- | --- | --- |
|  |  |  | eaf | β | se | P-value | eaf | β | se | *P*-value |
| rs10116520 | G | A | 0.400404 | 0.071981 | 0.010571 | 9.81E-12 | 0.401846 | -0.00075 | 0.009305 | 0.935623 |
| rs10269874 | T | G | 0.153302 | 0.07823 | 0.014143 | 3.18E-08 | 0.153551 | 0.024468 | 0.012586 | 0.05188 |
| rs10774624 | A | G | 0.599817 | -0.05914 | 0.010598 | 2.40E-08 | 0.599164 | 0.014822 | 0.009326 | 0.111998 |
| rs1391373 | C | T | 0.413927 | 0.128887 | 0.010483 | 9.64E-35 | 0.413862 | -0.01796 | 0.009263 | 0.052481 |
| rs2079672 | A | G | 0.604472 | -0.07258 | 0.010626 | 8.49E-12 | 0.603652 | 0.009592 | 0.009343 | 0.304571 |
| rs3744374 | A | G | 0.229396 | -0.08896 | 0.012531 | 1.26E-12 | 0.228946 | -0.00417 | 0.01085 | 0.700758 |
| rs3939286 | C | T | 0.762241 | -0.14461 | 0.011893 | 5.12E-34 | 0.761636 | 0.013062 | 0.010671 | 0.220938 |
| rs41260844 | T | C | 0.299921 | -0.07146 | 0.011452 | 4.37E-10 | 0.30005 | 0.011052 | 0.00995 | 0.266694 |
| rs4402589 | G | T | 0.499428 | 0.058 | 0.01039 | 2.37E-08 | 0.499751 | 0.022049 | 0.009108 | 0.01549 |
| rs4803791 | A | G | 0.303327 | -0.0628 | 0.011457 | 4.23E-08 | 0.303606 | -0.00602 | 0.009956 | 0.545544 |
| rs584768 | A | G | 0.410777 | 0.079087 | 0.010501 | 5.01E-14 | 0.410816 | -0.0072 | 0.009253 | 0.436673 |
| rs6889889 | A | G | 0.30049 | 0.123317 | 0.011157 | 2.12E-28 | 0.300783 | 0.001487 | 0.009928 | 0.880936 |
| rs6967330 | A | G | 0.268474 | 0.069528 | 0.011692 | 2.73E-09 | 0.268871 | -0.00473 | 0.010322 | 0.6469 |
| rs773107 | G | A | 0.279122 | 0.06907 | 0.011541 | 2.16E-09 | 0.278804 | 0.036624 | 0.010173 | 0.000318 |
| rs887972 | A | G | 0.292745 | 0.069657 | 0.011324 | 7.68E-10 | 0.293663 | -0.008 | 0.009983 | 0.422778 |
| rs919000 | A | G | 0.790373 | 0.072242 | 0.013002 | 2.76E-08 | 0.790574 | 0.050069 | 0.01135 | 1.03E-05 |
| rs9271715 | T | C | 0.296112 | -0.09758 | 0.01153 | 2.59E-17 | 0.295994 | 0.011431 | 0.010015 | 0.25371 |
| rs962992 | C | G | 0.303245 | -0.08418 | 0.011419 | 1.68E-13 | 0.302747 | -0.01121 | 0.009915 | 0.258275 |

Supplementary Figure 1


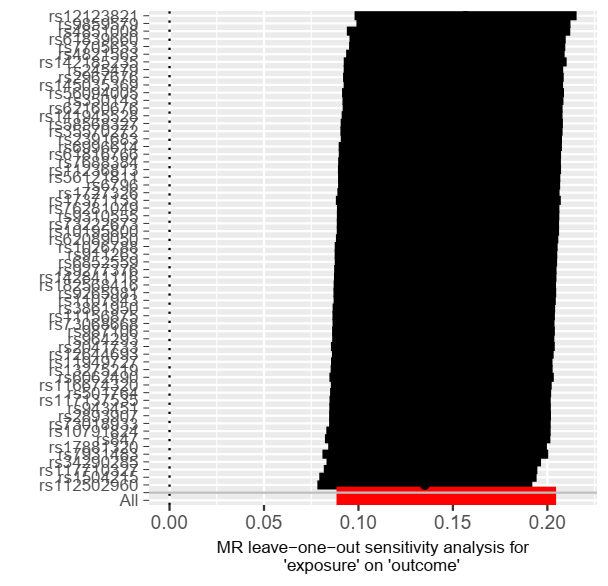


Supplementary Figure 2
